# Estimation of secondary cancer risk after radiotherapy in high‐risk prostate cancer patients with pelvic irradiation

**DOI:** 10.1002/acm2.12972

**Published:** 2020-07-16

**Authors:** Emel Haciislamoglu, Gorkem Gungor, Gokhan Aydin, Emine Canyilmaz, Ozan Cem Guler, Ahmet Yasar Zengin, Kamil Mehmet Yenice

**Affiliations:** ^1^ Department of Radiation Oncology Faculty of Medicine Karadeniz Technical University Trabzon Turkey; ^2^ Department of Radiation Oncology Acibadem University Istanbul Turkey; ^3^ Department of Radiation Oncology Faculty of Medicine Baskent University Adana Turkey; ^4^ Department of Radiation Oncology Kanuni Research and Education Hospital Trabzon Turkey; ^5^ Department of Radiation and Cellular Oncology University of Chicago Medicine Chicago IL USA

**Keywords:** flattening filter free (FFF), prostate cancer, radiation‐induced secondary cancer, radiotherapy (RT), volumetric modulated arc therapy (VMAT)

## Abstract

We aimed to estimate the risk of secondary cancer after radiotherapy (RT) in high‐risk prostate cancer (HRPC) patients with pelvic irradiation. Computed tomography data of five biopsy‐proven HRPC patients were selected for this study. Two different planning target volumes (PTV_1_ and PTV_2_) were contoured for each patient. The PTV_1_ included the prostate, seminal vesicles, and pelvic lymphatics, while the PTV_2_ included only the prostate and seminal vesicles. The prescribed dose was 54 Gy for the PTV_1_ with a sequential boost (24 Gy for the PTV_2_). Intensity‐modulated RT (IMRT) and volumetric modulated arc therapy (VMAT) techniques were used to generate treatment plans with 6 and 10 MV photon energies with the flattening filter (FF) or flattening filter‐free (FFF) irradiation mode. The excess absolute risks (EARs) were calculated and compared for the bladder, rectum, pelvic bone, and soft tissue based on the linear‐exponential, plateau, full mechanistic, and specific mechanistic sarcoma dose‐response model. According to the models, all treatment plans resulted in similar risks of secondary bladder or rectal cancer and pelvic bone or soft tissue sarcoma except for the estimated risk of the bladder according to the full mechanistic model using IMRT_(6MV;FF)_ technique compared with VMAT techniques with FFF options. The overall estimation of EAR indicated that the radiation‐induced cancer risk due to RT in HRPC was lower for bladder than the rectum. EAR values ranged from 1.47 to 5.82 for bladder and 6.36 to 7.94 for rectum, depending on the dose–response models used. The absolute risks of the secondary pelvic bone and soft tissue sarcoma were small for the plans examined. We theoretically predicted the radiation‐induced secondary cancer risk in HRPC patients with pelvic irradiation. Nevertheless, prospective clinical trials, with larger patient cohorts with a long‐term follow‐up, are needed to validate these model predictions.

## 1 INTRODUCTION

Prostate cancer is the second most common malignancy in men in the world.^1^ Radiotherapy (RT) plays an essential role in nearly every stage of the prostate cancer. According to the European Association of Urology Guidelines, intensity‐modulated RT (IMRT), with long‐term androgen deprivation therapy, is the main treatment option for high‐risk prostate cancer (HRPC) patients.^2^ Prophylactic pelvic nodal irradiation has failed to show beneficial results in cN0 patients.^3^ The STAMPEDE trial time to failure‐free survival was worse in patients with N+ disease (HR, 2.02 [95% confidence interval (CI), 1.46–2.81]) than in those with N0 disease.^4^ Hence, the pelvic lymph node irradiation is still a confounding problem for clinicians, and it is important to carefully weigh the relative advantage of adding the pelvic lymph node irradiation over its potential toxicity or increased secondary cancer risk in the HRPC patients.

The development of secondary cancers after RT is an undesired outcome of the therapy that can be observed in long‐term cancer survivors. There are several factors that impact secondary cancer risk which can be analysed from a radiobiological perspective: age at irradiation, type of irradiated tissue, irradiated volume, treatment technique, previous irradiation/radiological investigations.^5^ In this study, secondary cancer risks were assessed using Schneider's concept of the organ equivalent dose (OED) and excess absolute risk (EAR). The EAR of developing secondary cancer after exposure to radiation can be estimated from the organ specific differential dose–volume histograms (dDVHs) based on biological models that are fitted to data from atomic bomb survivors and Hodgkin patients treated with RT.^6^, ^7^, ^8^, ^9^, ^10^

To our knowledge, no previous report has estimated radiation‐induced secondary cancer risks in HRPC patients with pelvic irradiation. The study aimed to estimate radiation‐induced secondary cancer risks using pelvic irradiation in HRPC patients in modern RT techniques with different energy levels and flattening filter (FF) options.

## 2 MATERIALS AND METHODS

### 2.A. Treatment planning volumes

**Fig. 1:**
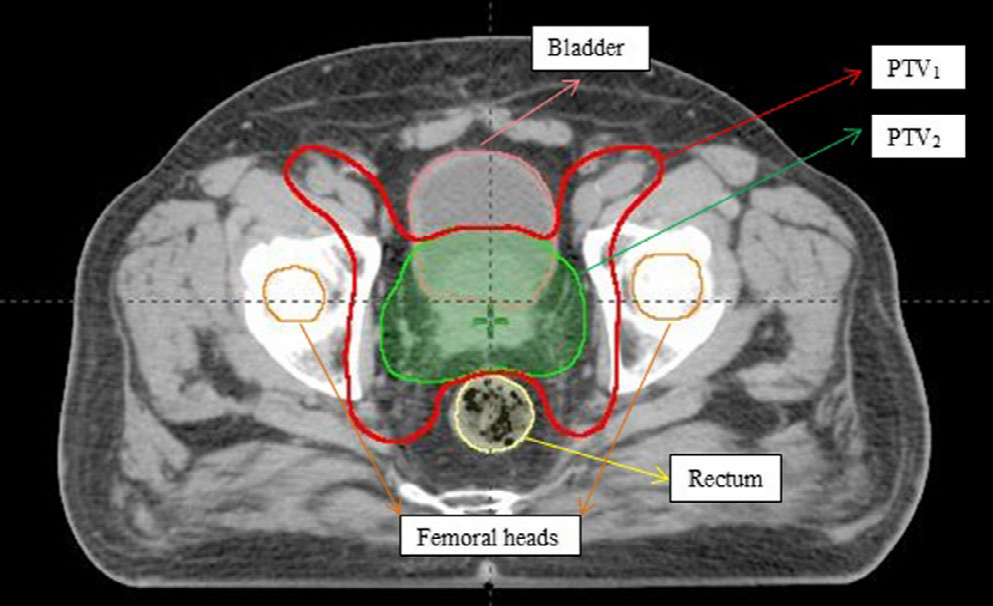
An example of the contour of the planning target volumes (PTVs) and organs at risk (OARs) on the axial plane.

Computed tomography data of five biopsy‐proven HRPC patients was randomly selected from our database for a retrospective planning study. Two different planning target volumes (PTV_1_ and PTV_2_) were delineated for each patient. The PTV_1_ included the prostate, seminal vesicles, and pelvic lymphatics, while PTV_2_ included only the prostate and seminal vesicles (Fig. 1). The mean PTV_1_ and PTV_2_ volumes of five patients were 1022.30 and 278.94 cm^3^ respectively. The bladder, rectum, and femoral heads were considered organs at risk (OAR) (Fig. 1). In addition, all pelvic bones were contoured, and pelvic soft tissue was created as a structure (total scanned volume in the irradiation field minus all pelvic bones and PTVs).

### 2.B. Treatment planning

IMRT and volumetric modulated arc therapy (VMAT) techniques were used to generate treatment plans. The prescribed dose was 54 Gy in 27 fractions for the PTV_1_ with a sequential boost (24 Gy in 12 fractions for the PTV_2_). At least 95% of the PTVs were required to receive ≥95% of the prescription dose. The dose–volume constraints used for the OARs are listed in Table 1.

**Table 1:** Dose constraints of the organs at risk (OARs).

OAR	Goal or constraint dose
Rectum	V_70_ ≤ 20%
	V_50_ ≤ 50%
Bladder	V_70_ ≤ 30%
	V_55_ ≤ 50%
Femoral heads	V_50_ < 5%

In total, five different treatment plans with 6 and 10 MV photon energies were created for each patient, including IMRT plans with FF and VMAT plans with FF and flattening filter‐free (FFF) irradiation modes. Herein, the plans are referred to as IMRT_(6MV;FF)_, VMAT_(6MV;FF)_, VMAT_(6MV;FFF)_, VMAT_(10MV;FF)_, and VMAT_(10MV;FFF)_.

While the IMRT plans consisted of 11 equally spaced coplanar beams, the VMAT plans used three full coplanar arcs. All plans were optimized in the Eclipse treatment planning system (version13.6, Varian Medical Systems) using the identical optimization parameters for the targets and OARs. Once each plan was optimized, the dDVH's for the bladder, rectum, pelvic bone, and pelvic soft tissue were extracted and used to calculate the OEDs and EARs for each plan type and for all study cases.

### 2.C. Calculation of second malignancy risk estimates

The risk of developing a secondary solid cancer after RT is usually represented by the EAR. The EAR to develop solid cancer describes the absolute difference in cancer rates of persons exposed to a dose d and those not exposed to a dose beyond the natural dose exposition per 10.000 persons per year (PY).^9^ The EAR can be calculated according to Eq. (1):

(1) $$EAR\left(D,e,a\right)=EAR_{0}\cdot RED_{D}\cdot \mu \left(agex,agea\right)$$

where $EAR_{0}$ is the initial slope of the dose–response curve at a low dose and the RED (risk equivalent dose) is the dose–response relationship for radiation‐induced cancer in units of dose. The function μ takes into account the age of the population examined, based on the patient’s age at the time of irradiation (agex), and the attained age of the patient in years (agea). The factor μ is given by Eq. (2):

(2) $$\mu \left(agex,agea\right)=\exp\left[\gamma_{e}\cdot \left(agex‐30\right)+\gamma_{a}\cdot ln\left(agea/70\right)\right]$$

where $\gamma_{e}$ and $\gamma_{a}$ are age modifying factors ($EAR_{0}$ was originally calculated for persons exposed at age 30 and attaining age 70). All EAR calculations in this study were calculated for patients irradiated at age 60 (agex) and attaining age 80 (agea) as representing the HRPC population at risk.

The EAR, as defined in Eq. (1), is the mathematically modeled EAR in a small volume element of an organ or tissue. If the dose–volume histogram of an organ of interest is known, the EAR for that organ (EAR_org_) can be obtained using the Eq. (1) by a convolution of the dose–volume histogram with the EAR by Eq. (3):

(3) $$EAR_{\mathrm{org}}=\frac{1}{V_{T}}\sum_{i}V_{\mathrm{Di}}EAR_{0}RED_{\mathrm{Di}}\mu \left(agex,agea\right)$$

where $V_{T}$ is the total organ volume. The sum is taken over all the bins of the dose–volume histogram, and $V_{\mathrm{Di}}$ is the volume of the organ that is exposed to dose $D_{i}$_._

It is known, that for doses below 2 Gy, the dose–response relation is linear.^11^ For higher doses and inhomogeneous dose distributions, however, the dose–response relation is no longer linear^6^, ^11^ and other dose–response functions are required to describe their relation. To facilitate the estimation of secondary cancer risks in the irradiated organs, Schneider et al.^6^, ^7^ introduced the concept of the OED which assumes that any two dose distributions in an organ are equivalent if they cause the same radiation‐induced cancer incidence.^6^ The OED is then described by the following expression in Eq. (4):

(4) $$OED=\frac{1}{V_{T}}\sum_{i}V_{\mathrm{Di}}RED_{\mathrm{Di}}$$

and the $EAR_{\mathrm{org}}$ can be estimated as in Eq. (5):

(5) $$EAR_{\mathrm{org}}=EAR_{0}\cdot OED\cdot \mu \left(agex,agea\right)$$

The OED values are independent of the $EAR_{0}$ and modifying function µ.^9^ The $EAR_{0}$ values for Western populations and different sites were taken from the study by Schneider et al.^9^ and tabulated in Table 2. There are different models for the RED calculation, based on different assumptions of cells’ behavior after dose exposition.^7^, ^9^

**Table 2:** Parameters used in the secondary malignancy risk calculation.

Site	EAR_0_	Full mechanistic model		Linear‐exponential model	Plateau model	Age modifying factors	
		ά (Gy^−1^)	R	ά(Gy^‐1^)	ά(Gy^−1^)	γ_e_	γ_a_
Bladder	3.8	0.219	0.06	0.213	0.633	−0.024	2.38
Rectum	0.73	0.033	0.56	0.031	0.065	−0.024	2.38
Pelvic bone (Sarcoma Model): R = 0.5; ά (Gy^−1^):0.067; EAR_0_: 0.20						−0.013	−0.56
Pelvic soft tissue (Sarcoma Model): R = 0.5; ά (Gy^−1^):0.060; EAR_0_: 0.60						−0.013	−0.56

The linear model assumes a linear response over the whole dose range:

(6) $$RED_{D}=D$$

The mechanistic model that accounts for killing and fractionation effects:^8^, ^9^

(7) $$RED_{D}=\frac{e^{‐\overset{\prime }{\alpha }D}}{\overset{\prime }{\alpha }R}\left(1‐2R+R^{2}e^{\overset{\prime }{\alpha }D}‐{\left(1‐R\right)}^{2}e^{‐\frac{\overset{\prime }{\alpha }R}{1‐R}D}\right)$$

where $\overset{\prime }{\alpha }=\alpha +\beta \frac{D}{D_{T}}d_{T}$

The parameters α and β are those from the linear quadratic model of cell killing, describing the linear and quadratic dose–response of the tissue to radiation. The parameter R describes the repopulation and ability to repair between dose fractions delivered.

For this model, there are two limiting cases with the parameter R = 0 for no repair and R = 1 for full repopulation/repair occurring. Therefore, the limit R = 0 leads to the linear‐exponential model:

(8) $$RED_{D}=De^{‐\overset{\prime }{\alpha }D}$$

The linear exponential model takes into account that the probability for cell killing increases exponentially with dose which leads to a decrease of the risk of cancer induction due to the killing of mutated cells.

Moreover, the limit R = 1 is the case of full repopulation/repair, known as the plateau model:

(9) $$RED_{D}=\frac{1‐e^{‐\overset{\prime }{\alpha }D}}{x}$$

Previous studies have demonstrated that, for inhomogeneous dose distributions above 4 Gy, the full mechanistic, plateau, and linear‐exponential models represent a better description of the dose–response function than the linear model.^7^, ^9^ Therefore, the linear model was not included in our results.

OEDs for the bladder and rectum were calculated for the three different dose–response models based on the dDVHs according to:

(10) $$OED_{\mathrm{mechanistic}}=\frac{1}{V_{T}}\sum_{i}V_{\mathrm{Di}}\frac{e^{‐\overset{\prime }{\alpha }D_{i}}}{\overset{\prime }{\alpha }R}\left(1‐2R+R^{2}e^{\overset{\prime }{\alpha }D_{i}}‐{\left(1‐R\right)}^{2}e^{‐\frac{\overset{\prime }{\alpha }R}{1‐R}D_{i}}\right)$$

(11) $$OED_{linear‐exp}=\frac{1}{V_{T}}\sum_{i}V_{\mathrm{Di}}D_{i}e^{‐\overset{\prime }{\alpha }D_{i}}$$

(12) $$OED_{\mathrm{plateau}}=\frac{1}{V_{T}}\sum_{i}V_{\mathrm{Di}}\frac{1‐e^{‐\overset{\prime }{\alpha }D_{i}}}{\overset{\prime }{\alpha }}$$

The risk of radiation‐induced pelvic bone and soft tissue secondary malignancies were calculated using a specific mechanistic sarcoma model based on an intermediate repopulation (R = 0.5) according to:

(13) $$OED_{\mathrm{sarcoma}}=\frac{1}{V_{T}}\sum_{i}V_{\mathrm{Di}}\frac{e^{‐\overset{\prime }{\alpha }D_{i}}}{\overset{\prime }{\alpha }R}\left(1‐2R+R^{2}e^{\overset{\prime }{\alpha }D_{i}}‐\overset{\prime }{\alpha }RD_{i}‐{\left(1‐R\right)}^{2}e^{‐\frac{\overset{\prime }{\alpha }R}{1‐R}D_{i}}\right)$$

The site‐specific parameters were derived from a combined fit to data from atomic bomb survivors and Hodgkin patients treated with single doses of 2–40 Gy assuming an α/β value of 3 Gy.^9^ The difference between the baseline risks of developing cancer, without exposure to radiation for the Japanese and Western populations was included. The parameters used for the EAR calculations are shown in Table 2.

### 2.D. Statistical analysis

The statistical analysis was performed using SPSS software (version 18.0). Quantitative data are expressed as the mean ± standard deviation. Multiple groups of means were compared with a one‐way analysis of variance, after testing for variance equality. Variance homogeneity was assessed using Levene's test. A *post‐hoc* test was used for situations where there were significant differences between groups.

## 3 RESULT

The OED and EAR values for the bladder, rectum, pelvic bone and soft tissue calculated with different dose–response models are shown in Tables 3 and 4. The tabulated data shows values averaged over all patients. Average EARs for all three dose–response models for the bladder and rectum were 3.15 and 7.59 respectively for IMRT_(6MV;FF)_, 3.32 and 7.01 respectively for VMAT_(6MV;FF)_, 3.35 and 6.85 respectively for VMAT_(6MV;FFF)_, 3.32 and 6.92 respectively for VMAT_(10MV;FF)_, and 3.35 and 6.83 respectively for VMAT_(10MV;FFF)_.

**Table 3:** Organ equivalent dose (OED) for the bladder, rectum, pelvic bone, and soft tissue.

Plan	Bladder			Rectum			Pelvic bone	Soft tissue
	OED_lin‐exp_	OED_plat_	OED_mech_	OED_lin‐exp_	OED_plat_	OED_mech_	OED_sarcoma_	OED_sarcoma_
IMRT_(6MV;FF)_	0.79 ± 0.13	3.13 ± 0.05	**1.16 ± 0.11**	19.64 ± 1.43	21.81 ± 2.02	22.19 ± 2.12	3.20 ± 0.26	1.23 ± 0.15
VMAT_(6MV;FF)_	0.95 ± 0.09	3.12 ± 0.06	1.29 ± 0.07	18.04 ± 1.63	20.49 ± 3.18	20.28 ± 2.49	3.28 ± 0.24	1.29 ± 0.12
VMAT_(6MV;FFF)_	0.97 ± 0.08	3.12 ± 0.07	1.31 ± 0.07	17.81 ± 1.58	19.67 ± 2.24	20.00 ± 2.36	3.24 ± 0.30	1.27 ± 0.15
VMAT_(10MV;FF)_	0.94 ± 0.07	3.12 ± 0.07	1.29 ± 0.06	18.00 ± 1.67	19.84 ± 2.39	20.17 ± 2.51	3.24 ± 0.25	1.20 ± 0.12
VMAT_(10MV;FFF)_	0.97 ± 0.06	3.11 ± 0.07	1.31 ± 0.06	17.77 ± 1.77	19.61 ± 2.41	19.93 ± 2.54	3.18 ± 0.26	1.15 ± 0.13

The mean values and standard deviation of the OED averaged over all patients. The bold value indicate a significantly lower OED compared to VMAT_(6MV;FFF)_ and VMAT_(10MV;FFF)_. Abbreviations: IMRT, intensity‐modulated radiotherapy; VMAT, volumetric modulated arc therapy; FF, flattening filter; FFF, flattening filter free; lin‐exp, linear‐exponential; plat, plateau; mech, full mechanistic; and sarcoma specific mechanistic sarcoma dose–response model. OED unit is Gray.

**Table 4:** The excess absolute risk (EAR) for the bladder, rectum, pelvic bone, and soft tissue.

Plan	Bladder			Rectum			Pelvic bone	Soft tissue
	EAR_lin‐exp_	EAR_plat_	EAR_mech_	EAR_lin‐exp_	EAR_plat_	EAR_mech_	EAR_sarcoma_	EAR_sarcoma_
IMRT_(6MV;FF)_	1.47 ± 0.25	5.82 ± 0.08	**2.16 ± 0.20**	7.03 ± 0.51	7.80 ± 0.72	7.94 ± 0.76	0.43 ± 0.04	0.50 ± 0.06
VMAT_(6MV;FF)_	1.76 ± 0.16	5.81 ± 0.11	2.40 ± 0.14	6.45 ± 0.59	7.33 ± 1.14	7.26 ± 0.89	0.45 ± 0.03	0.53 ± 0.05
VMAT_(6MV;FFF)_	1.80 ± 0.16	5.81 ± 0.12	2.44 ± 0.13	6.37 ± 0.56	7.04 ± 0.80	7.15 ± 0.85	0.44 ± 0.04	0.52 ± 0.06
VMAT_(10MV;FF)_	1.75 ± 0.13	5.80 ± 0.12	2.40 ± 0.11	6.43 ± 0.60	7.10 ± 0.85	7.22 ± 0.90	0.44 ± 0.04	0.49 ± 0.05
VMAT_(10MV;FFF)_	1.81 ± 0.11	5.79 ± 0.13	2.44 ± 0.11	6.36 ± 0.61	7.01 ± 0.86	7.13 ± 0.91	0.43 ± 0.04	0.47 ± 0.05

The mean values and standard deviation of the EAR averaged over all patients. The bold value indicate a significantly lower EAR value compared with VMAT_(6MV;FFF)_ and VMAT_(10MV;FFF)_. Abbreviations: IMRT, intensity‐modulated radiotherapy; VMAT, volumetric modulated arc therapy; FF, flattening filter; FFF, flattening filter free; lin‐exp, linear‐exponential; plat plateau, mech full mechanistic, and sarcoma specific mechanistic sarcoma dose–response model. EAR unit is per 10 000 persons per year.

When comparing the plans, IMRT_(6MV;FF)_ significantly reduced the OED and EAR of the bladder, according to the full mechanistic model, compared to VMAT_(6MV;FFF)_ and VMAT_(10MV;FFF)_ (*P*‐values 0.036, 0.030, 0.031, and 0.030, respectively), despite resulting in similar risks of secondary bladder cancer based on the linear‐exponential and plateau dose–response model. There were no statistically significant differences between the plans, in all techniques, beam energies, and irradiation modes with regard to EAR of the rectum, pelvic bone, and soft tissue, regardless of the model used.

The relative differences in the EAR between the plans predicted using the linear–exponential, plateau, and full mechanistic dose–response models were lower for the rectum compared with the bladder. Within each model, absolute differences between individual plans were small. The absolute risks of the pelvic bone and soft tissue sarcoma were small for the plans examined. The EAR ranges for all techniques were 0.43–0.45 for the pelvic bone and 0.47–0.53 for the soft tissue (Table 4).

**Fig. 2:**
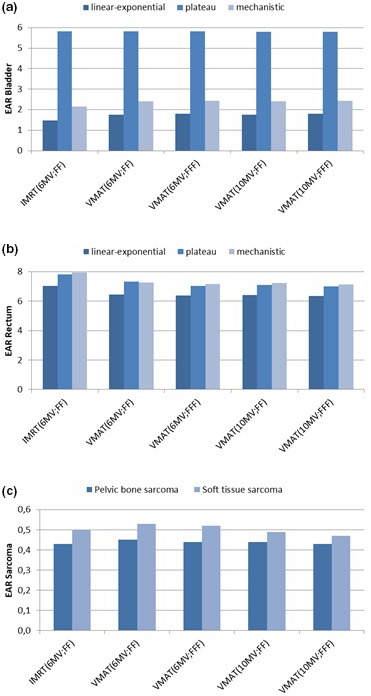
The excess absolute risk (EAR) of the bladder, rectum, pelvic bone and, soft tissue. The EAR based on differential dose–volume histograms of (a) the bladder, (b) rectum, (c) pelvic bone, and soft tissue for pelvic irradiation. Calculation of the EAR was performed using the linear‐exponential (dark blue), plateau (blue), and full mechanistic (light blue) dose–response models for the bladder and rectum. The EAR calculation was performed using the sarcoma model for the pelvic bone (dark blue) and soft tissue (blue). The mean values per 10 000 PY per Gy averaged over all five patients are shown. IMRT intensity‐modulated radiotherapy, VMAT volumetric modulated arc therapy, FF flattening filter, FFF flattening filter free.

When comparing FFF plans with the equivalent FF plans, for the bladder, rectum, pelvic bone, and soft tissue, there were minimal differences in the EAR. The relative differences of the EAR for each organ type, all the treatment techniques and all dose–response model are plotted in Fig. 2.

## 4 DISCUSSION

Radiation‐induced secondary malignancies is one of the important late side effects of RT. Many factors may contribute to the development of radiation‐induced secondary malignancies, such as the age at the time of RT, the dose and volume of the irradiated area, type of the irradiated organ and tissue, radiation technique and individual and family history of cancer. Theoretical concerns have been raised regarding a potentially large increase in secondary cancer risk using modern techniques such as IMRT compared to three‐dimensional conformal RT (3D‐CRT).^11^, ^12^, ^13^, ^14^ However, the direct clinical evidence of increased secondary cancers from the IMRT over 3DCRT is still lacking since IMRT is relatively new technology in the clinic with about <20 yr of wide use in clinics.^15^, ^16^

Age at the time of radiation exposure is one of the main factors involved in radiation‐induced cancer. Whereas the cancer risks due to radiation exposures in childhood have been extensively documented in the literature,^17^, ^18^, ^19^, ^20^ the relationship between radiation‐induced cancer risk and age at exposure in adulthood is less clear. Epidemiological data from Japanese atomic bomb survivors and children exposed to radiation for medical reasons suggest that excess relative risks for radiation‐induced cancers at a given attained age are substantially higher for individuals who are exposed during childhood than for those exposed at older ages.^20^, ^21^, ^22^, ^23^, ^24^ However, the weight of epidemiological evidence now suggests that, for adult exposures, radiation‐induced cancer risks do not generally decrease with increasing age at exposure.^25^, ^26^, ^27^ In the Life Span Study cohort of the Japanese atomic bomb survivors, the excess relative risks for radiation‐induced cancers as a function of age at exposure were examined.^27^ As expected, the excess relative risk for cancer induction was higher during childhood and decreased progressively at exposure ages of 30–40. Surprisingly, the excess relative risk of developing solid tumors ramped up again for exposure ages higher than 40 years old.^27^ In addition, Richardson et al. observed that the radiation doses received after the age of 45 showed a stronger association with cancer mortality than those received at younger ages.^28^ All these findings suggest that radiation sensitivity, measured in terms of carcinogenic events, increases with age among adults after the age of 40–45. Therefore, prostate cancer is a disease of relatively older patient but also it can be considered as a suitable group for the risk estimation of secondary cancer.

The secondary malignancy estimation after RT is becoming an important subject for comparative treatment planning. The data from modern treatment planning systems provide accurate, three dimensional dose distributions for each individual patient, thereby opening up new possibilities for more precise estimates of secondary cancer incidence rates in the irradiated organs. While radiation‐induced secondary cancer risk from IMRT has been compared to that from 3D‐CRT in prostate planning studies,^14^, ^29^, ^30^, ^31^, ^32^ far fewer comparisons with other techniques, such as VMAT^33^ and FFF techniques,^34^, ^35^ have been performed. To our knowledge, no previous report has estimated radiation‐induced secondary cancer risks in HRPC patients with pelvic irradiation.

Radical RT for prostate cancer offers excellent long‐term outcomes for patients with high‐risk disease. The increased risk of pelvic nodal involvement in this cohort has led to the development of whole‐pelvis RT with a prostate boost. The clinical evidence suggests that the pelvic irradiation and non‐pelvic irradiation techniques with hormonotherapy result in equally efficacious outcomes for RT.^3^ There have been many publications on this subject, yet it is still unclear. In the current, pelvic irradiation with the addition of hormonotherapy is still a more common treatment option available to patients with HRPC in routine practice. Therefore, while deciding to use pelvic irradiation as a treatment regimen, estimation of the risk of secondary malignancy is important. We aimed to estimate radiation‐induced secondary cancer risk following modern prostate RT techniques for HRPC patients with pelvic irradiation, by using the concepts of OED and EAR with multiple dose–response models.

IMRT with long‐term androgen deprivation therapy is the main treatment option for HRPC patients.^2^ However, the use of VMAT has grown in the recent years and become a common choice for treatment of prostate cancer, as treatment planning studies have shown comparable or decreased dose to OAR and reduced treatment time when compared to IMRT.^36^

Secondary cancer risks following IMRT of the prostate were predicted in previous studies.^29^, ^32^, ^37^, ^38^ Fontenot et al.^38^ found that the bladder and rectum carried the greatest predicted risk of secondary cancer. According to Murray *et al.,* ^37^ the rectal and bladder cancer EARs ranged 1.44–2.69 and 1.70–2.42 per 10 000 PY, respectively, using the mechanistic model for early PC patients, using the 3D‐CRT, IMRT, VMAT with FF and FFF beams, and stereotactic ablative radiotherapy. The results from our study for the rectal and bladder cancer EARs ranged 7.13–7.94 and 2.16–2.44 per 10 000 PY, respectively, using the mechanistic model. Direct comparison of our data with data from other groups is not straightforward because of the difference in irradiation volumes. Their irradiated volumes were not reported but it is obvious that they were smaller than ours, as it was the early prostate cancer patients with non‐pelvic irradiation that were analyzed in their study. The non‐pelvic irradiation plans delivered lower doses to the OAR compared to pelvic irradiation plans due to the smaller volume of the PTV. As a result, the lower doses to the OARs contributed to the reduction of all calculated EARs with respect to the pelvic irradiation technique.

In the present study, we showed the radiation‐induced secondary cancer risks of pelvic irradiation in all organs involved in the treatment of HRPC for a specific age group in this patient population according to different dose–response models. When comparing techniques, IMRT‐PI_(6MV;FF)_ plan significantly reduced the secondary cancer risk of the bladder according to the full mechanistic model compared with VMAT plans with FFF options*,* even though the absolute risks of secondary bladder cancer were small for the plans examined. This result may be attributed to the fact that the mechanistic model includes repair and repopulation effects.^8^, ^9^ As for the rectum, unlike the bladder, the secondary cancer risk in HRPC patients was independent of the treatment techniques, energies, modes, and dose–response models used. Additionally, this study showed that the absolute risks of the secondary pelvic bone and soft tissue sarcoma were small for the plans examined.

Regardless of the risk model selected, our findings revealed that VMAT did not increase the predicted risk when compared to IMRT, despite VMAT distributing lower dose over a larger volume of normal tissue than IMRT.^39^, ^40^

We showed that the FFF beam plans resulted in very small differences in EARs compared with the equivalent FF beam plans, for the bladder, rectum, pelvic bone, and soft tissue in our study. This finding agrees well with the results by Murray et al.^37^ However, their results showed some statistically significant differences between the FFF and FF beams for calculated secondary cancer risks in the organs outside of the treatment fields.

There are a number of limitations in our work. We performed this analysis for the secondary cancer risks for only five patients selected from our patient data base. Other studies on similar cancer risk assessments also used a small number of patient cases, typically 2–3 cases per study. The reason for a relatively small sample size in this type of study is that the primary interest is to investigate the differences between the planning techniques rather than the factors due to inter‐patient variability.^30^, ^32^, ^37^, ^38^, ^39^, ^40^, ^41^, ^42^ There are also other uncertainties in radiation‐induced secondary cancer models and parameters. Schneider's concept of OED was employed as this incorporates the fractionation, and repair and repopulation (with the mechanistic model).^9^ Models based on the full and no repair/repopulation, were also adopted to illustrate a range of possibilities for estimating the secondary cancer risk factors. The results of this study show secondary cancer risks for all the OARs from pelvic irradiation in HRPC with three different dose–response models. However, we did not assess the impact of hormonal therapy on secondary cancer risks for the HRPC patients, even though it is accepted that the systemic cancer treatment with chemotherapy and hormonal therapy is also associated with increased risk of secondary malignant neoplasm.^43^ Another aspect of relevance for the cancer risks from modern RT techniques is the use of image‐guidance techniques employing ionizing radiation. Risk evaluations accounting simultaneously for primary radiation and imaging doses have shown that the additional risk due to repeated imaging is in fact very small.^44^ Such doses were not available for the patients used retrospectively for this study, and therefore we did not include the impact of image‐guided radiotherapy on secondary cancer risk. Finally, we did not attempt to estimate the secondary cancer risks in out‐of‐field organs. In contrast with our study which looked into only in‐field or close‐to‐the field organs and tissues, there appears to be a real difference between the plans using the FFF beams versus FF beams with the FFF beams resulting in plans with smaller calculated risk factors in out‐of‐field organs.^34^, ^35^, ^36^, ^37^ This is a reasonable and somewhat straightforward expectation since there is no flattening filter material in the beam path for a FFF beam, therefore the FFF beam produces less scatter in the beam production that results in less deposited dose to the organs outside of the direct treatment field. Despite this difference, the calculated absolute risk benefits from the FFF at large distances from the irradiated area, were very small for prostate cancer patients in the age range we considered in our study. Nevertheless, treatment of younger patients would have possibly resulted in greater absolute benefits from the FFF beams.

In our study, we attempted to minimize different planning system software and treatment margins since both may contribute to different secondary cancer risk estimates by creating plans, using the same planning system, and using the same dose constraint and PTV margin.

## 5 CONCLUSION

A major strength of this study is its novelty; to our knowledge, this was the first study to predict the risk of secondary cancer incidence following RT in HRPC patients with pelvic irradiation. We compared radiation‐induced secondary cancer risks using several contemporary, clinically relevant RT techniques such as IMRT and VMAT. This study demonstrated the radiation‐induced secondary cancer risks of pelvic irradiation in all organs involved in the treatment of HRPC for a specific age group in this patient population according to different dose–response models. Patient‐specific considerations like the irradiated and attaining age of the patient or the addition of adjuvant hormone therapy may influence these findings. Until any clinical data regarding radiation‐induced secondary cancers in HRPC patients treated with pelvic irradiation is obtained, data from this study can be of importance in the process of informing individual patients or obtaining consent from them.

## CONFLICT OF INTEREST

The authors declare that there is no conflict of interest regarding the publication of this article.

## References

[1] Bray F , Ferlay J , Soerjomataram I , Siegel RL , Torre LA , Jemal A . Global cancer statistics 2018: GLOBOCAN estimates of incidence and mortality worldwide for 36 cancers in 185 countries. CA Cancer J Clin. 2018;68:394–424. 30207593 10.3322/caac.21492

[2] Mottet N , Bellmunt J , Bolla M , et al. Guidelines on prostate cancer. Part 1: screening, diagnosis, and local treatment with curative intent. Eur Urol. 2017;71:618–629. 27568654 10.1016/j.eururo.2016.08.003

[3] Lawton CA , DeSilvio M , Roach M 3rd , et al. An update of the phase III trial comparing whole pelvic to prostate only radiotherapy and neoadjuvant to adjuvant total androgen suppression: updated analysis of RTOG 94–13, with emphasis on unexpected hormone/radiation interactions. Int J Radiat Oncol Biol Phys. 2007;69:646–655. 17531401 10.1016/j.ijrobp.2007.04.003 PMC2917177

[4] James ND , Spears MR , Clarke NW , et al. Failure‐free survival and radiotherapy in patients with newly diagnosed nonmetastatic prostate cancer: data from patients in the control arm of the STAMPEDE trial. JAMA Oncol. 2016;2:348–357. 26606329 10.1001/jamaoncol.2015.4350 PMC4789485

[5] Marcu LG . Photons ‐ radiobiological issues related to the risk of second malignancies. Phys Med. 2017;42:213–220. 28236557 10.1016/j.ejmp.2017.02.013

[6] Schneider U , Zwahlen D , Ross D , Kaser‐Hotz B . Estimation of radiation‐induced cancer from three‐dimensional dose distributions: concept of organ equivalent dose. Int J Radiat Oncol Biol Phy. 2005;61:1510–1515. 10.1016/j.ijrobp.2004.12.040 15817357

[7] Schneider U , Walsh L . Cancer risk estimates from the combined Japanese A‐bomb and Hodgkin cohorts for doses relevant to radiotherapy. Radiat Environ Biophys. 2008;47:253–263. 18157543 10.1007/s00411-007-0151-y

[8] Schneider U . Mechanistic model of radiation‐induced cancer after fractionated radiotherapy using the linear‐quadratic formula. Med Phys. 2009;36:1138–1143. 19472619 10.1118/1.3089792

[9] Schneider U , Sumila M , Robotka J . Site‐specific doseresponse relationships for cancer induction from the combined Japanese A‐bomb and Hodgkin cohorts for doses relevant to radiotherapy. Theor Biol Med Model. 2011;8:27. 21791103 10.1186/1742-4682-8-27 PMC3161945

[10] Schneider U , Sumila M , Robotka J , Gruber G , Mack A , Besserer J . Dose‐response relationship for breast cancer induction at radiotherapy dose. Radiat Oncol. 2011;6:67. 21651799 10.1186/1748-717X-6-67 PMC3127785

[11] Hall EJ , Wuu C‐S . Radiation‐induced second cancers: the impact of 3D‐CRT and IMRT. Int J Radiat Oncol Biol Phys. 2003;56:83–88. 12694826 10.1016/s0360-3016(03)00073-7

[12] Followill D , Geis P , Boyer A . Estimates of whole‐body dose equivalent produced by beam intensity modulated conformal therapy. Int J Radiat Oncol Biol Phys. 1997;38:667–672. 9231693 10.1016/s0360-3016(97)00012-6

[13] Kry SF , Followill D , White RA , Stovall M , Kuban DA , Salehpour M . Uncertainty of calculated risk estimates for secondary malignancies after radiotherapy. Int J Radiat Oncol Biol Phys. 2007;68:1265–1271. 17637398 10.1016/j.ijrobp.2007.04.014

[14] Stathakis S , Li J , Ma CC . Monte Carlo determination of radiation‐induced cancer risks for prostate patients undergoing intensity‐modulated radiation therapy. J Appl Clin Med Phys. 2007;8:2685. 18449157 10.1120/jacmp.v8i4.2685 PMC5722626

[15] Huang J , Kestin LL , Ye H , Wallace M , Martinez AA , Vicini FA . Analysis of second malignancies after modern radiotherapy versus prostatectomy for localized prostate cancer. Radiother Oncol. 2011;98:81–86. 20951450 10.1016/j.radonc.2010.09.012

[16] Zelefsky MJ , Housman DM , Pei X , et al. Incidence of secondary cancer development after high‐dose intensity‐modulated radiotherapy and image‐guided brachytherapy for the treatment of localized prostate cancer. Int J Radiat Oncol Biol Phys. 2012;83:953–959. 22172904 10.1016/j.ijrobp.2011.08.034

[17] Cardis E , Kesminiene A , Ivanov V , et al. Risk of thyroid cancer after exposure to 131I in childhood. J Natl Cancer Inst. 2005;97:724–732. 15900042 10.1093/jnci/dji129

[18] Kleinerman RA . Cancer risks following diagnostic and therapeutic radiation exposure in children. Pediatr Radiol. 2006;36:121–125. 16862418 10.1007/s00247-006-0191-5 PMC2663653

[19] Little MP . Leukaemia following childhood radiation exposure in the Japanese atomic bomb survivors and in medically exposed groups. Radiat Prot Dosimetry. 2008;132:156–165. 18936088 10.1093/rpd/ncn264

[20] Preston DL , Cullings H , Suyama A , et al. Solid cancer incidence in atomic bomb survivors exposed in utero or as young children. J Natl Cancer Inst. 2008;100:428–436. 18334707 10.1093/jnci/djn045

[21] Preston DL , Shimizu Y , Pierce DA , Suyama A , Mabuchi K . Studies of mortality of atomic bomb survivors. Report 13: solid cancer and noncancer disease mortality: 1950–1997. Radiat Res. 2003;160:381–407. 12968934 10.1667/rr3049

[22] Walsh L . Heterogeneity of variation of relative risk by age at exposure in the Japanese atomic bomb survivors. Radiat Environ Biophys. 2009;48:345–347. 19479272 10.1007/s00411-009-0229-9

[23] Little MP . Heterogeneity of variation of relative risk by age at exposure in the Japanese atomic bomb survivors. Radiat Environ Biophys. 2009;48:253–262. 19471953 10.1007/s00411-009-0228-x

[24] Little MP , De Vathaire F , Charles MW , Hawkins MM , Muirhead CR . Variations with time and age in the risks of solid cancer incidence after radiation exposure in childhood. Stat Med. 1998;17:1341–1355. 9682324 10.1002/(sici)1097-0258(19980630)17:12<1341::aid-sim852>3.0.co;2-6

[25] Shuryak I , Hahnfeldt P , Hlatky L , Sachs RK , Brenner DJ . A new view of radiation‐induced cancer: integrating short‐ and long‐term processes. Part II: second cancer risk estimation. Radiat Environ Biophys. 2009;48:275–286. 19499238 10.1007/s00411-009-0231-2 PMC2714894

[26] Shuryak I , Hahnfeldt P , Hlatky L , Sachs RK , Brenner DJ . A new view of radiation‐induced cancer: integrating short‐ and long‐term processes. Part I: approach. Radiat Environ Biophys. 2009;48:263–274. 19536557 10.1007/s00411-009-0230-3 PMC2714893

[27] Shuryak I , Sachs RK , Brenner DJ . Cancer risks after radiation exposure in middle age. J Natl Cancer Inst. 2010;102:1628–1636. 20975037 10.1093/jnci/djq346 PMC2970575

[28] Richardson DB , Wing S . Greater sensitivity to ionizing radiation at older age: follow‐up of workers at Oak Ridge National Laboratory through 1990. Int J Epidemiol. 1999;28:428–436. 10405844 10.1093/ije/28.3.428

[29] Kry SF , Salehpour M , Followill DS , et al. The calculated risk of fatal secondary malignancies from intensity‐modulated radiation therapy. Int J Radiat Oncol Biol Phys. 2005;62:1195–1203. 15990025 10.1016/j.ijrobp.2005.03.053

[30] Kry SF , Salehpour M , Followill DS , et al. Out‐of‐field photon and neutron dose equivalents from step‐and‐shoot intensity‐modulated radiation therapy. Int J Radiat Oncol Biol Phys. 2005;62:1204–1216. 15990026 10.1016/j.ijrobp.2004.12.091

[31] Ruben JD , Davis S , Evans C , et al. The effect of intensity modulated radiotherapy on radiation‐induced second malignancies. Int J Radiat Oncol Biol Phys. 2008;70:1530–1536. 18207670 10.1016/j.ijrobp.2007.08.046

[32] Bednarz B , Athar B , Xu XG . A comparative study on the risk of second primary cancers in out‐of‐field organs associated with radiotherapy of localized prostate carcinoma using Monte Carlo based accelerator and patient models. Med Phys. 2010;37:1987–1994. 20527532 10.1118/1.3367012 PMC2862056

[33] Rechner LA , Howell RM , Zhang R , Etzel C , Lee AK , Newhauser WD . Risk of radiogenic second cancers following volumetric modulated arc therapy and proton arc therapy for prostate cancer. Phys Med Biol. 2012;57:7117–7132. 23051714 10.1088/0031-9155/57/21/7117 PMC3772654

[34] Kry SF , Vassiliev ON , Mohan R . Out‐of‐field photon dose following removal of the flattening filter from a medical accelerator. Phys Med Biol. 2010;21:2155–2166. 10.1088/0031-9155/55/8/003 20305334

[35] Halg RA , Besserer J , Schneider U . Systematic measurements of whole‐body dose distributions for various treatment machines and delivery techniques in radiation therapy. Med Phys. 2012;39:7662–7676. 23231314 10.1118/1.4767773

[36] Teoh M , Clark CH , Wood K , Whitaker S , Nisbet A . Volumetric modulated arc therapy: a review of current literature and clinical use in practice. Br J Radiol. 2011;84:967–996. 22011829 10.1259/bjr/22373346 PMC3473700

[37] Murray LJ , Thompson CM , Lilley J , et al. Radiation‐induced second primary cancer risks from modern external beam radiotherapy for early prostate cancer: impact of stereotactic ablative radiotherapy (SABR), volumetric modulated arc therapy (VMAT) and flattening filter free (FFF) radiotherapy. Phys Med Biol. 2015;60:1237–1257. 25590229 10.1088/0031-9155/60/3/1237

[38] Fontenot JD , Lee AK , Newhauser WD . Risk of secondary malignant neoplasms from proton therapy and intensity‐modulated x‐ray therapy for early‐stage prostate cancer. Int J Radiat Oncol Biol Phys. 2009;74:616–622. 19427561 10.1016/j.ijrobp.2009.01.001 PMC4120808

[39] Kjaer‐Kristoffersen F , Ohlhues L , Medin J , Korreman S . RapidArc volumetric modulated therapy planning for prostate cancer patients. Acta Oncol. 2009;48:227–232. 18855157 10.1080/02841860802266748

[40] Zhang P , Happersett L , Hunt M , Jackson A , Zelefsky M , Mageras G . Volumetric modulated arc therapy: planning and evaluation for prostate cancer cases. Int J Radiat Oncol Biol Phys. 2010;76:1456–1462. 19540062 10.1016/j.ijrobp.2009.03.033

[41] Schneider U . Calculated risk of fatal secondary malignancies from intensity‐modulated radiotherapy: in regard to Kry et al (letter). Int J Radiat Oncol Biol Phys. 2006;64:1290. 16504771 10.1016/j.ijrobp.2005.10.032

[42] Blais AR , Lederer E , Oliver M , Leszczynski K . Static and rotational step‐and‐shoot IMRT treatment plans for the prostate: a risk comparison study. Med Phys. 2012;39:1069–1078. 22320817 10.1118/1.3679338

[43] Morton LM , Onel K , Curtis RE , Hungate EA , Armstrong GT . The rising incidence of second cancers: patterns of occurrence and identification of risk factors for children and adults. Am Soc Clin Oncol Educ Book. 2014;2014:57–67. 10.14694/EdBook_AM.2014.34.e57 24857148

[44] Ardenfors O , Josefsson D , Dasu A . Are IMRT treatments in the head and neck region increasing the risk of secondary cancers? Acta Oncol. 2014;53:1041–1047. 24983652 10.3109/0284186X.2014.925581

